# On the impact of Citizen Science-derived data quality on deep learning based classification in marine images

**DOI:** 10.1371/journal.pone.0218086

**Published:** 2019-06-12

**Authors:** Daniel Langenkämper, Erik Simon-Lledó, Brett Hosking, Daniel O. B. Jones, Tim W. Nattkemper

**Affiliations:** 1 Biodata Mining Group, Faculty of Technology, Bielefeld University, Bielefeld, Germany; 2 National Oceanography Centre, University of Southampton Waterfront Campus, Southampton, United Kingdom; New York University School of Medicine, UNITED STATES

## Abstract

The evaluation of large amounts of digital image data is of growing importance for biology, including for the exploration and monitoring of marine habitats. However, only a tiny percentage of the image data collected is evaluated by marine biologists who manually interpret and annotate the image contents, which can be slow and laborious. In order to overcome the bottleneck in image annotation, two strategies are increasingly proposed: “citizen science” and “machine learning”. In this study, we investigated how the combination of citizen science, to detect objects, and machine learning, to classify megafauna, could be used to automate annotation of underwater images. For this purpose, multiple large data sets of citizen science annotations with different degrees of common errors and inaccuracies observed in citizen science data were simulated by modifying “gold standard” annotations done by an experienced marine biologist. The parameters of the simulation were determined on the basis of two citizen science experiments. It allowed us to analyze the relationship between the outcome of a citizen science study and the quality of the classifications of a deep learning megafauna classifier. The results show great potential for combining citizen science with machine learning, provided that the participants are informed precisely about the annotation protocol. Inaccuracies in the position of the annotation had the most substantial influence on the classification accuracy, whereas the size of the marking and false positive detections had a smaller influence.

## Introduction

In recent years computer vision has made a big leap forward in tackling some of the most demanding problems such as detection of cars or people in photos, owing to the emergence of deep learning [1, 2]. Deep learning methods for image classification and object detection were successfully proposed but mostly limited to everyday image domains, i.e. images showing “everyday objects” from human civilization such as cars, furniture, people. Please note that in machine learning, especially in deep learning, these images are often referred to as natural images, but due to the possibility of misunderstandings in interdisciplinary research, we will refer to them as everyday images. The employment of deep learning algorithms brings with it a number of requirements and assumptions, i) availability of huge collections of annotated image data, ii) good image quality (high signal-to-noise-ratio, no extreme light exposure, limited cast shadows) and iii) high pixel-resolution for objects of interest in the images. One reason for the rapid progress of deep learning in computer vision is the availability of many large image collections (see i) above), accumulated by internet-based projects (e.g. ImageNet [3]). Because everyday images are of common interest, much research focuses on these image collections. Object detection/segmentation/classification contests like ILSVRC (ImageNet Large Scale Visual Recognition Competition [4]) are held to compare network performance. Furthermore, the competitors publish the pre-trained models, online, providing a starting point for future projects. Unfortunately, in marine science, there are no such datasets or contests available, which is likely also because the data volume is huge but the number of annotated images is very limited, i.e. labels describing the content on a semantic level. The only competition known to the authors is the *National Data Science Bowl—Predict ocean health, one plankton at a time* [5], but this competition is limited to plankton and did not receive the same amount of attention as, e.g. the ILSVRC did.

Marine image informatics is an emerging field settled at the intersection of marine biology and ecology, image processing, and machine learning. Owing to the availability of digital camera systems and underwater imaging platforms like Remote Operated Vehicles (ROVs), Autonomous Underwater Vehicles (AUVs) and towed sleds, large image collections (approx. 10^3^ − 10^4^ images) can be acquired during a single dive [6]. While the worldwide volume of available marine image data is huge and continuously growing, the employment of deep learning for marine image analysis is by far not straightforward, because of the following reasons:

1. There is usually not enough training data (see i)—above) because image annotation in marine sciences is non-trivial and laborious. Morphological/taxonomic classification is an *n*-class problem and requires a considerable amount of education, training and experience. Unlike in everyday images, where almost all objects can be recognized by almost everyone growing up in a modern civilization, marine objects of interest (such as fishes, sea cucumbers, starfishes) can be hard to detect and classify even for human experts, henceforth called experts for brevity. Therefore, image annotation performed by experts is time-consuming, error-prone and expensive, particularly as there are a limited number of experts available [7].
2. The annotation task is further complicated by the high diversity and low abundance of marine life in the deep sea [8]. A large number of classes are represented by a low number of examples. Moreover, it is a common observation that 80%-90% of the data belong to a small subset of *L*′ classes among the total number of *L* observed classes, with *L*′ ≪ *L*. This observation is called the data imbalance problem in machine learning classification tasks.
3. Also, the requirements ii) image quality and iii) pixel resolution are often not fulfilled satisfactorily. ROVs, AUVs, and towed sleds are rarely used just for the purpose of image acquisition. These platforms often carry other payloads and a multitude of sensor arrays serving different purposes. Even if only images are acquired, quite often there are multiple objectives, such as biodiversity studies, mapping the seafloor, resource assessment and habitat mapping. Therefore, the speed of the platform and the optimal distance to the seafloor can vary considerably. As a consequence, the object resolution and image quality may not be ideal, in contrast to everyday images, where objects usually take up 30%-70% of the image pixels. In many scenarios, the images are recorded below 200 m depth and so there is no natural light, therefore a flash has to be used. This leads to lighting artifacts, such as vignetting or illuminating marine snow (small particles in the water column are illuminated by the flash).

Marine image annotation can be described as a combination of two tasks, the task of object detection and the task of morphological/taxonomic classification. Object detection can be modeled as a one-class classification problem. In our recent work, we have shown that given enough training data, training of a deep learning model for marine image patch classification is possible [9, 10]. However, only the task of morphological/taxonomic classification for manually detected image patches was considered, the problem of object detection has not yet been addressed. Two computational approaches to the marine object detection problem have been published [11, 12], but no study about the generalization performance was reported yet, i.e. the applicability of these methods on an arbitrary marine dataset. In a recent study we proposed MAIA (Machine learning Assisted Image Annotation [13]), which shows promise in solving some of the object detection problems. However, it is unfit for certain scenarios, where a rather homogenous background is not given.

Fully automatic detection of objects in marine image collections may seem unfeasible in many scenarios. Here, we demonstrate a promising alternative to a computational approach: citizen science, which has quickly gained popularity in recent years [14]. In the context of image analysis, citizen scientists (CS) are usually non-domain experts performing a detection or classification task with a web-application. Some recent works analyze the use of CS to increase the power of deep learning classification [15]. In the context of this work, the CS would be asked to perform the task of general object detection, i.e. finding all interesting objects without focusing on a single object class as most CS annotation studies do.

**Fig 1:**
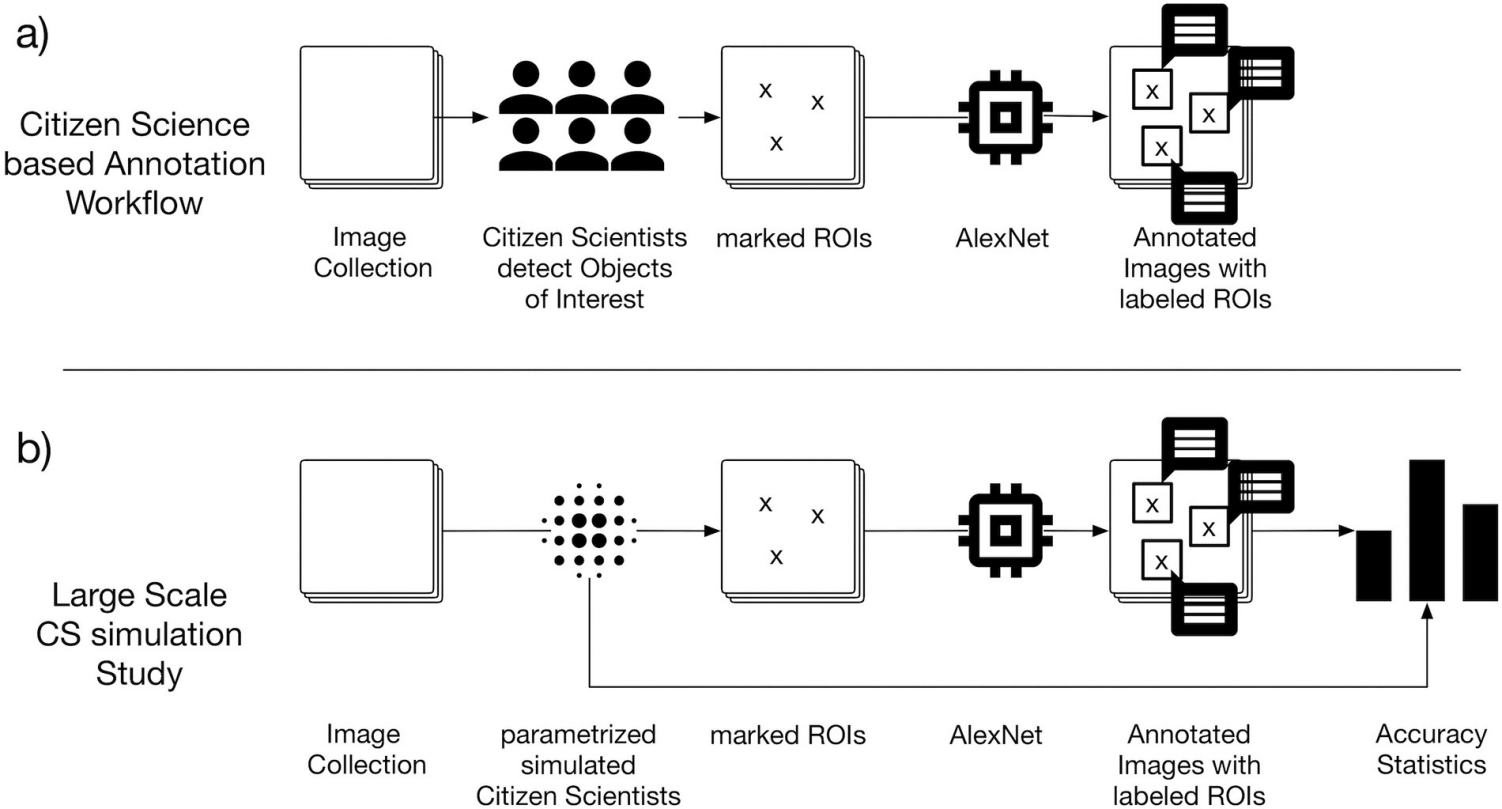
a) Workflow of a Citizen Science based annotation study. b) Workflow of our parametrized simulated Citizen Science study.

The above observations motivate a new strategy for the detection and classification of objects in marine images. First, citizen scientists are screening the large image collections for regions of interest (ROI) and mark those with a circle of adjustable size. In parallel a subset of the data is evaluated by an experienced domain expert, i.e. a marine biologist, who assigns labels to objects, thereby providing a training set for a deep learning classifier. After the citizen scientists have marked all ROI in the complete image collection, the trained classifier is applied to all ROI for taxonomic/morphological classification (cf. Fig 1a)). This way the expensive domain expert time could be employed most efficiently.

In this paper, we investigate the potential of such error-prone citizen science object detections in combination with powerful deep learning classifiers. In the first step I), we conducted a primer-experiment as a small test experiment for citizen science-based marine image analysis. The images were chosen to reflect typical examples and use cases. This primer-experiment gave us valuable insights into typical errors made by citizen scientists and protocol specifications for the main experiment. In step II), we performed a larger citizen science study including ten CS each of which manually evaluated the same set of ten images. The resulting data from I), II) and the gold standard annotations provided by an expert are used in order to simulate the outcome of a large-scale CS study in step III) resembling the errors usually done by CS. In step IV) we used the simulated annotations as input for a deep learning architecture trained on the expert gold standard annotations to answer the question if CS object detection is a valid/feasible replacement for expert object detections as a method to drive the attention of the trained deep learning classifier. By varying our simulation parameters, we were able to investigate the effects of different types of inaccuracies in the CS annotations, such as inaccurate position (IP) or inaccurate radius (IR) (cf. Fig 1b)).

## Materials and methods

### Images

**Fig 2:**
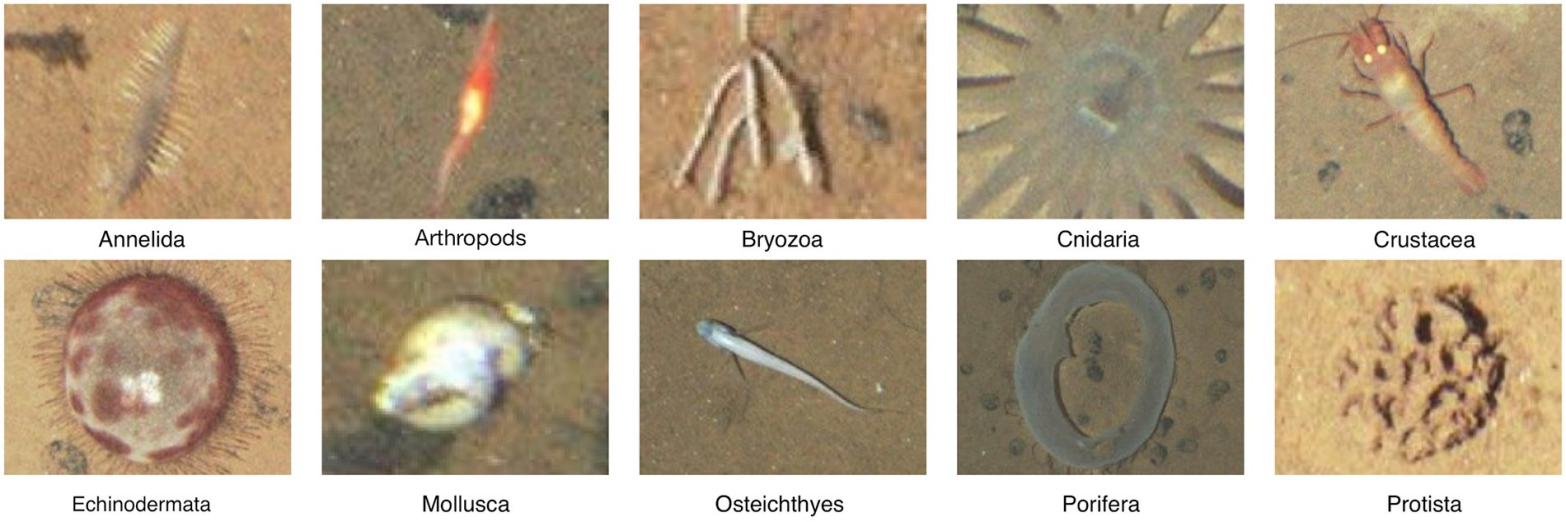
Example image patches for the object classes found in the APEI-6 area. Arthropods, *Mollusca*, *Echinodermata*, and also *Cnidaria* have a wide variety of shapes, but only one example image per label is shown for brevity.

The image collection {*I*_*n*,*n*=1…*N*_}, where *N* is the total number of images, used in this work is from a Pacific region referred to as the Area of Particular Environmental Interest 6 (APEI-6), centered on 122° 55’ W, 17° 16’ N. This is a marine protected area designed to safeguard and monitor biodiversity and ecosystem function in an abyssal Pacific region (the Clarion Clipperton Zone) targeted for deep-sea nodule mining [16]. The dataset comprises *N* = 10052 images of size 2448 × 2048 pixels with a total of *M* = 54894 annotations. The images were captured using a digital camera mounted to the Autosub 6000 AUV [17] at depths between 4013 m and 4235 m and with a distance to the seabed between 2 m and 4 m. Ten different classes are annotated by experts (for examples see Fig 2). In this paper, Arthropods is a morphotype, i.e. a category based on visual appearance rather than on taxonomy, encompassing a multitude of heterogeneous objects on a coarse level, as they cannot be labeled on a finer level due to image quality.

### Methods

Domain experts annotated a volume of images {*I*_*n*,*n*=1…*N*_}. An annotation is defined as tuple $A_{a}=\left(x,y,r,I_{n},\Omega \right)$, with *x* and *y* being the position of the center of a square, *r* being its radius (i.e. half of the side length), *I*_*n*_ being the Image on which the annotation is done and Ω being the label, i.e. morphotype/taxonomic category assigned to it. The set of all annotations $A=\{A_{a},a=0\ldots M\}$ is split into a training set T and a validation set V with the ratio 90%/10%, with T∪V=A and T∩V=∅.

#### I) Citizen science primer-experiment (CSP)

**Fig 3:**
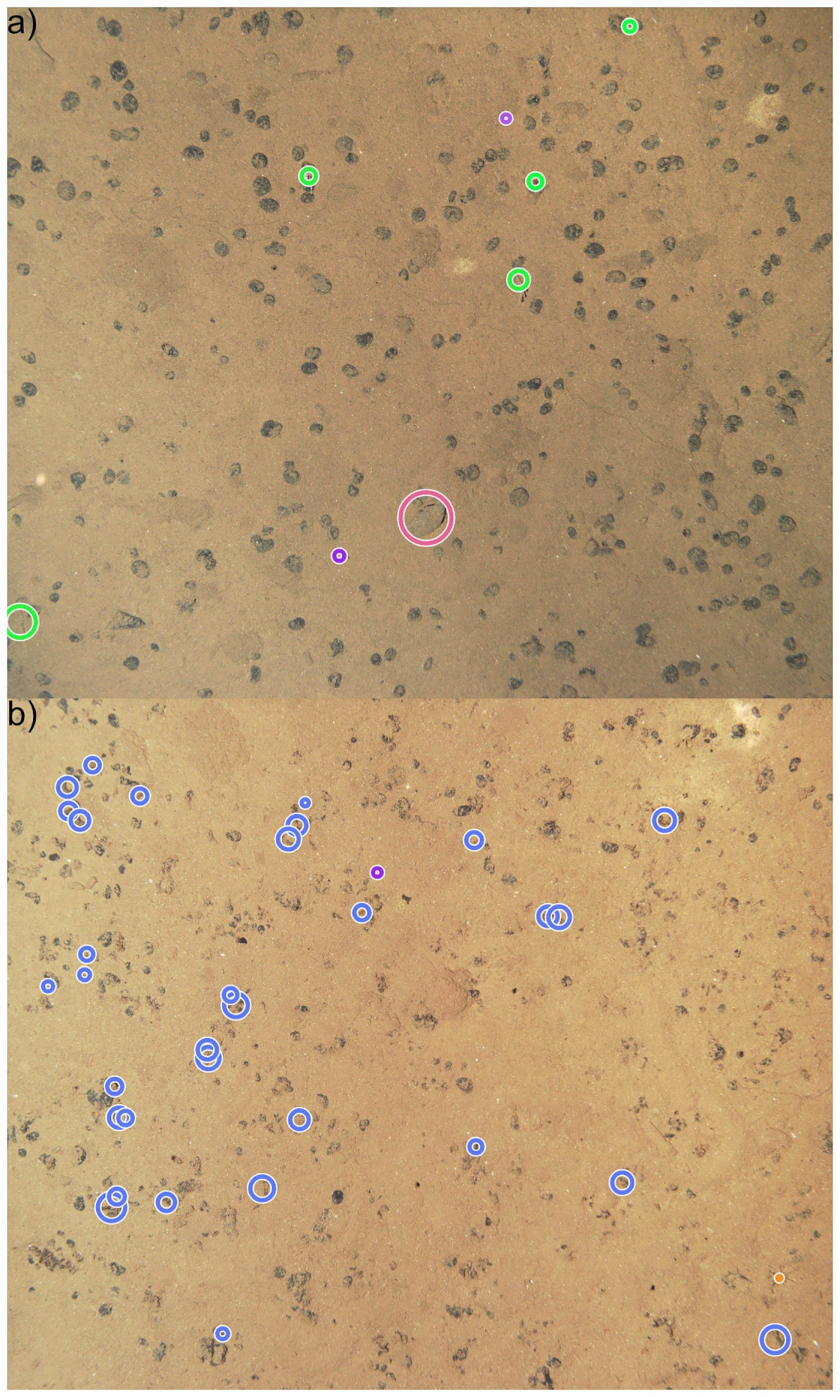
Images used for the CS primer-experiment including the expert annotations, whereas the color indicates the taxa/morphotype assigned by the expert. Image a) features larger easier to spot objects, while image b) mostly features hard to spot *Porifera*.

In the citizen science primer-experiment eight CS detected and marked objects in two hand-selected images. In contrast to the expert, the CS only needed to detect and mark objects without providing a label, thus a CS annotation is defined as a tuple $A^{C}=\left(x^{C},y^{C},r^{C},I_{n}\right)$. One image featuring visually easier to spot objects and one showing harder to spot small objects were presented. The annotations were gathered using the Biigle 2.0 [18] annotation system. The CS were given a video instruction on how to use the annotation tool and were provided with the example images shown in Fig 2, as well as the instruction to look for interesting objects (excluding stones/nodules and sand). Furthermore, the CS were advised to zoom in to identify smaller objects. Further instructions were not provided intentionally. One could, for instance, provide a specific protocol on the zoom level, or the specific part of each object that should be annotated, which would require the CS to additionally classify the object implicitly. We intentionally omitted this, because this would involve the classification of objects and the application of a specific protocol, which would lead to errors. In addition, we wanted to investigate whether relatively good results could be achieved without the use of a protocol. If this were possible, it would make the realization of a CS study easier and reduce the necessary effort, i.e. more people can be recruited without the need for preparation. We evaluated the CS annotations against the expert annotations A, depicted in Fig 3.

#### II) Citizen science study (CSS)

In the Citizen Science Study hereinafter referred to as CSS, we asked ten CS to annotate every image, i.e. provide CS annotations $A^{C}$, without a label to image regions, of a hand-selected set of ten images, so that each image was inspected by ten CS. Motivated by the observations in the primer-study we eliminated protocol differences by asking the expert and the CS to label like the intuitive CS protocol from the CSP experiment, i.e. encircling the whole object. The instructions to the CS and the execution of the study were identical to the CSP-experiment.

#### III) Simulated citizen scientists (SimCS)

Based on the observations in the primer-experiment CSP and the CS study CSS we simulated CS object detection for all images {*I*_*n*,*n*=1…*N*_}. This enabled us to assess the final classification results for these annotations, as we could use the domain expert’s original annotations as a gold standard. To render one set of simulated CS detections, the domain expert annotations $A=\{A_{a}\}$ were taken and transformed (*x*, *y*, *r*, *I*_*n*_, Ω) ↦ (*x*′, *y*′, *r*′, *I*_*n*_, Ω) to simulate inaccurate positioning (IP) or radius selection (IR) different from the expert’s annotation. In the following, we will omit the annotation index *a* for clarity of presentation. To simulate annotations suffering from inaccurate positioning (IP), we computed new artificial annotations $C_{p}^{o}=\left\{\left(x^{\prime },y^{\prime },r,I_{n},\Omega \right)\right\}$ resembling the behavior of citizen scientists, with *o* being the minimum overlap with the original annotation (*x*, *y*, *r*, *I*_*n*_, Ω) in percent. To create $C_{p}^{o}$ in the vicinity of the expert annotations featured in the validation set $V_{a}$, we sample new positions (*x*′, *y*′) according to the following equations,

$$x^{\prime }=x+Z_{x}*r,$$ (1)

$$y^{\prime }=y+Z_{y}*r$$ (2)

with

$$Z_{x}∼U\left(-\left(1-\sqrt{o}\right)*2,\left(1-\sqrt{o}\right)*2\right)$$

and

$$Z_{y}∼U\left(-\left(1-\sqrt{o}\right)*2,\left(1-\sqrt{o}\right)*2\right)$$

with U(a,b) being the uniform distribution on the interval [*a*, *b*]. We explicitly use only the validation set as a basis to generate new positions to prevent mixture of training and validation set, also known as data leakage. For evaluation, three different test sets with different minimum overlaps $C_{p}^{87.5},C_{p}^{75},C_{p}^{50}$ were generated. For each expert annotation in the validation set V four different citizen scientist annotations $C_{p}^{o}$ were sampled.

To simulate inaccurate setting of the annotation radius (IR), we generate further sets $C_{r}^{s}=\left\{\left(x,y,r^{\prime },I_{n},\Omega \right)\right\}$. These overestimate the radius by a maximum of 10% and 25% respectively ($C_{r}^{0.1},C_{r}^{0.25})$ or underestimate it by a maximum of 10% and 25% ($C_{r}^{-0.1},C_{r}^{-0.25})$, but keep the positions of the expert annotations.

$$r^{\prime }=\left(1+Z_{r}\right)*r,\;Z_{r}∼U\left(0,s\right)$$ (3)

In addition, we simulate false positives (FP) annotation produced by CS. Therefore for each image (of the validation set V), we generate two random background annotations of the size 64 × 64, which are non-overlapping with expert annotations, i.e.

$$A^{bg}=\left(x^{b},y^{b},64,I_{n},\Omega =Background\right)$$

with

$$d(\left(x^{b},y^{b}\right),\left(x,y\right))>r+64)$$

(and *d*(⋅, ⋅) the Euclidean metric), which form $C^{bg}$.

**Fig 4:**
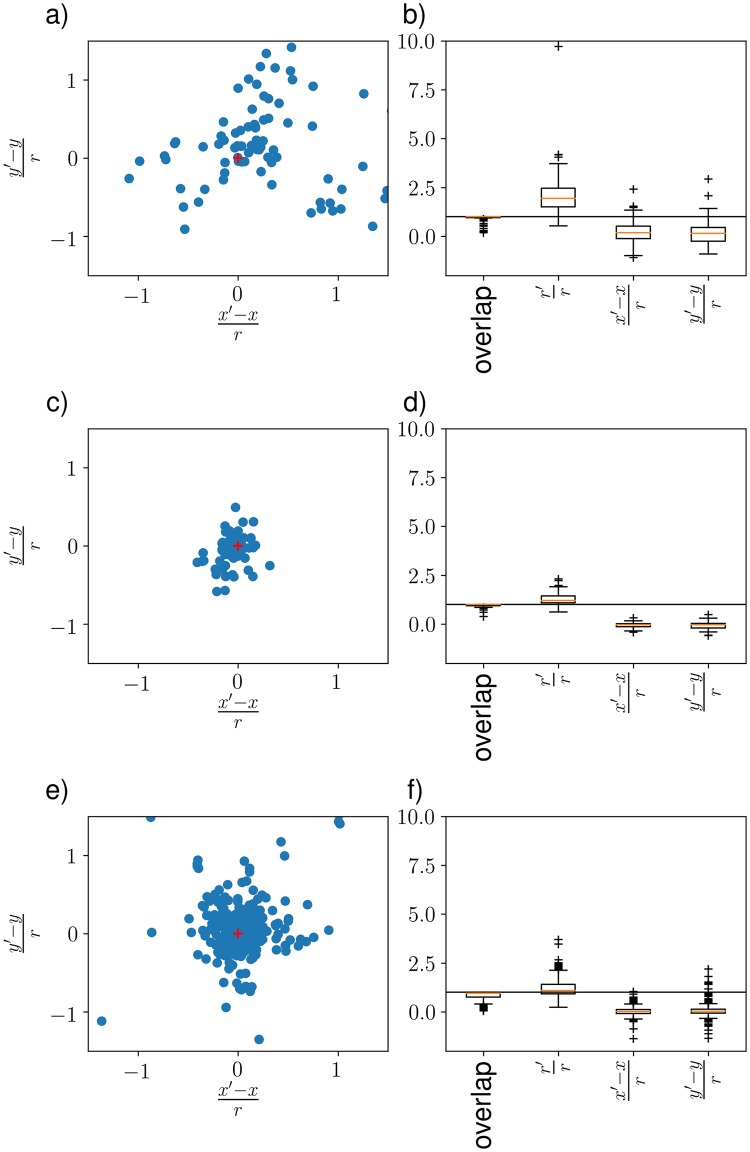
a) Deviation in x- and y-direction. Depicted are the deviation in the respective direction divided by the radius, i.e. $\frac{x^{\prime }-x}{r}$. Each blue dot represents a CS annotation $A^{C}$ of the Citizen Science Primer-experiment CSP. Please note that five outliers are not depicted in favor of a more detailed visualization. The red cross is just for orientation and depicts no deviation at all. b) Overlap displays the overlap of the expert annotation with the CS annotation. Relative radius $\frac{r^{\prime }}{r}$ is the ratio of the radius *r*′ of the CS annotation and the radius *r* of the expert annotation. c) and d) show the same information on the same scale as a) and b) respectively but are protocol-corrected (see section Results for more details). e) and f) show the results for the Citizen Science Study CSS. Each dot represents a CS annotation $A^{C}$ of the CSS..

Furthermore, we generated a dataset $C^{CSP}$ resembling the results of the CS primer-experiment CSP. Therefore we removed outliers and simulated deviations in position and radius using Gaussian distributions with parameters according to the CS primer-experiment data (see Fig 4b)).

**Fig 5:**
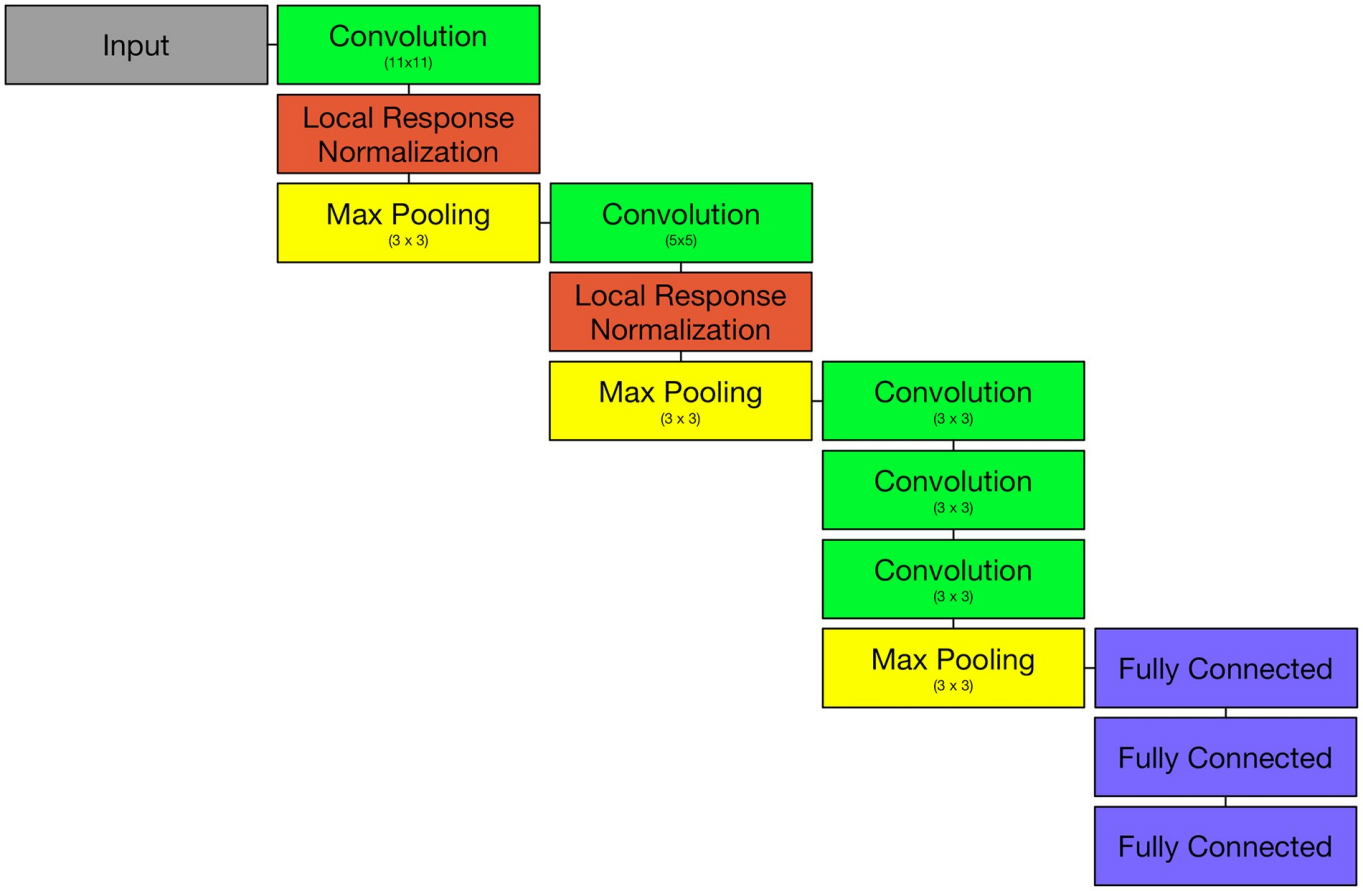
An overview of the modular architecture of AlexNet used for the classification of the (simulated) CS ROIs.

**Deep learning classifier** A classifier *f*(*x*) = Ω is trained on T. In this case, we used the well established AlexNet (see Fig 5) [19], because of its fast and robust classification. Training was performed using the caffe deep learning framework [20] and took 19 minutes on an NVIDIA Titan X. The network was trained for 30 epochs. No pretrained weights were used. Classifying the simulated data with the trained model took below 1 min for each dataset. The paper compares the results of f(V) and $f(C_{p}^{87.5})$, $f(C_{p}^{75})$, $f(C_{p}^{50})$, $f(C_{r}^{0.1})$, $f(C_{r}^{-0.1})$, $f(C_{r}^{0.25})$, $f\left(C_{r}^{-0.25}\right),f\left(C^{CSP}\right)$ as well as the ability to recognize background patches $f(C^{bg})$ in the following section.

### Ethics statement

In our image annotation experiment, no personal data was collected for individual users. And the individual performance/record of the users was not ranked or plotted or displayed. The evaluation of citizen scientist data was performed like it would have been performed for multiple annotators from the scientific community. Only a statistical evaluation was done, without paying attention to any individual performance. Verbal or written consent was given (depending on the way of communication) and recruitment was done by asking known non-scientific subjects.

## Results

Analyzing the CS primer-experiment from step I) we observed four kinds of errors or inaccuracies. First, the CS have produced false positives (FP), i.e. objects of no interest, or second, missed objects, so-called false negatives (FN). Third, in the case of true positive detections, the CS sometimes marked inaccurate positions (IP) of the annotations, or fourth, an inappropriate circle size (IR) was chosen.

The CS primer-experiment CSP produced 228 annotations {$A^{C}$} in total. Of these 82 (36%) were valid, i.e. had an overlap with one of the expert annotations {$A_{a}$}. This results in 146 (64%) false positives (FP). Of the 42 expert annotations, 21 (50%) were found and 21 (50%) were not found (FN). All of the 21 objects which were not found were of the same class (*Protista*). In the image featuring visually easier to spot objects, 8 out of 8 expert annotations were found. This shows how the quality of object detection is dependent on the difficulty of the task. There is a high deviation in the position of the CS annotations from the expert annotations in the CS primer experiment CSP (Fig 4a and 4b). It can be seen in Fig 4a) that most annotations are quite close to the expert annotations, but some large deviations can also be observed (cf. inaccurate position (IP)).

**Fig 6:**
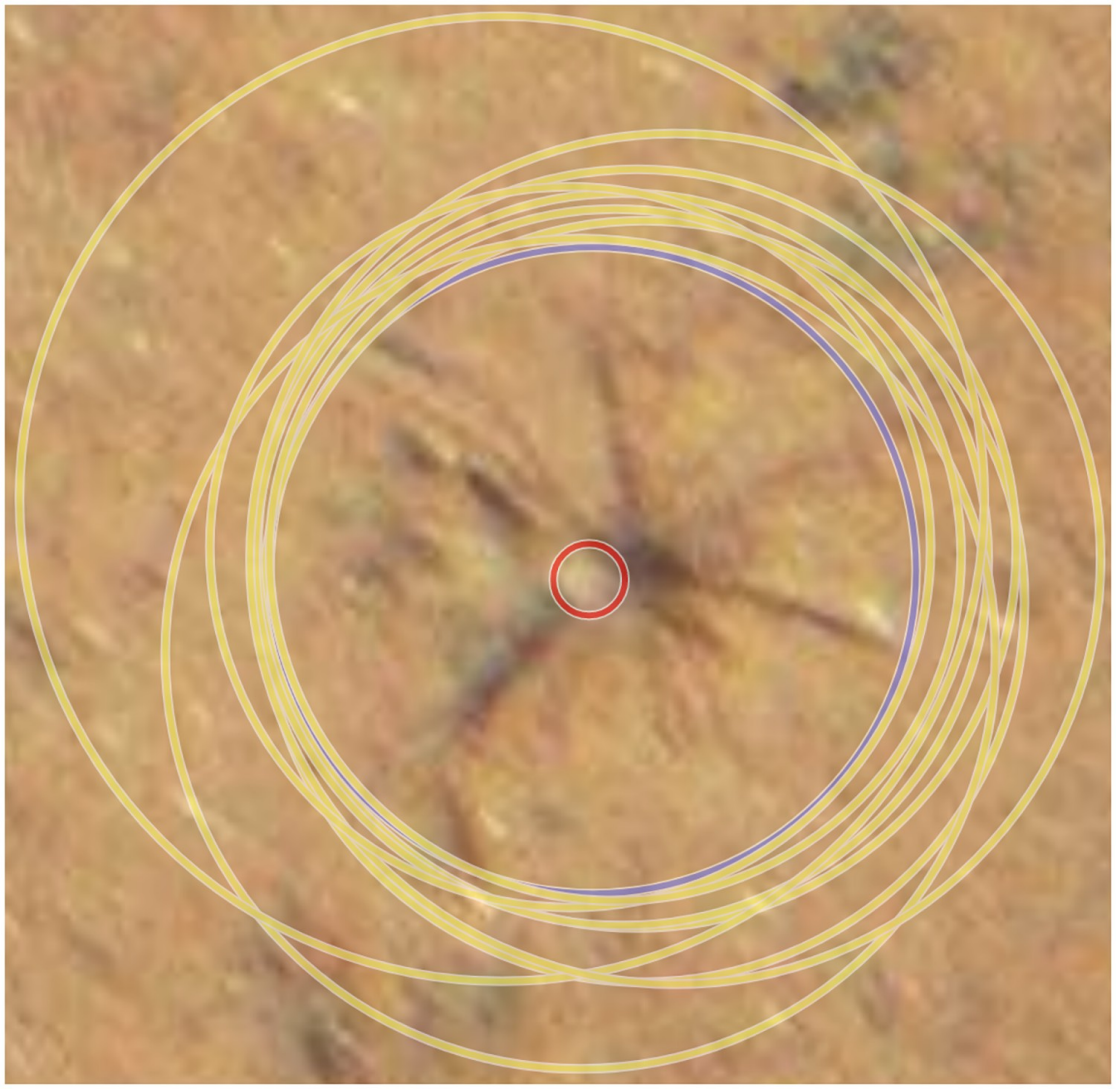
Results of the annotation of a *Porifera* in the CS primer-experiment CSP. The expert annotation is in red while the CS annotations are in yellow and the protocol-corrected expert annotation is in blue. The radii selected by the CS are more than 10 times the expert annotation radius.

Every person annotating image collections either implicitly or explicitly uses a protocol, i.e. a model on how to mark an object with a circle marker. The CS primer-experiment showed that the CS had implicitly used a protocol other than that of the expert. Experts marked objects not only to detect them but also to quantify their mass, thus sometimes only marking part of an object. In contrast, citizen scientists were focused on encircling the whole object, thus resulting in a change of center as well as a significantly higher radius (see Fig 6). In a second evaluation, we transformed the expert annotations {$A_{a}$} to the protocol-corrected expert annotations {$A_{a}^{\prime }$}. To this end the CS annotation protocol was applied to each expert annotation, i.e. the expert annotations were manually modified to enclose the entire object, also shifting the center of the annotation if necessary (cf. Fig 6). The results are shown in Fig 4c and 4d). The protocol differences can be confirmed comparing the CS annotations with the protocol-corrected expert annotations (cf. Fig 4a, 4b, 4c and 4d). The protocol differences are also observable when looking at the radii in Fig 4b and 4d), which are mostly overestimated (cf. inaccurate radius (IR)). Furthermore, we noticed that the CS rather increase the radius to encircle the whole object than running a risk of not capturing everything. This could also be owing to the lack of experience in operating annotation software on a regular basis, i.e. finding the correct center of an object as well as selecting an appropriate zoom level for the task (also cf. Fig 6). Owing to the rather generous radii provided by the CS annotators, most annotations have complete overlap with the expert annotations.

The CS study CSS produced 804 annotations {$A^{C}$} in total. Of these 366 (46%) were valid, i.e. had an overlap with expert annotations {$A_{a}$}. This results in 438 (54%) false positives (FP). Of the 108 expert annotations, 70 (65%) were found and 38 (35%) were not found (FN). In Fig 4e and 4f we show the resulting deviations of the expert annotations compared to the CS annotations as observed in the CS study CSS.

In Table 1 we show the classification accuracies for the different datasets starting with the validation data V (first column, Table 1).

**Table 1:** Classification accuracy for the expert, the simulated citizen scientist annotations, and the CS primer-experiment. The arrows are just for illustration purposes. Differences of more than 3 basis points compared to the results of V are depicted with ↓ or ↑. Differences of less than 3 basis points compared to V are depicted with ↘ or ↗.

		inaccurate position (IP)						inaccurate radius (IR)									
	V	$C_{p}87.5$		$C_{p}^{75}$		$C_{p}^{50}$		$C_{r}^{0.25}$		$C_{r}^{-0.25}$		$C_{r}^{0.1}$		$C_{r}^{-0.1}$		$C^{CSP}$	
*Protista*	0.84	↗	0.86	=	0.84	↓	0.72	↘	0.83	↗	0.87	=	0.84	↗	0.86	↓	0.55
*Porifera*	0.82	=	0.82	↘	0.79	↓	0.75	↘	0.81	↘	0.79	↗	0.84	↘	0.80	↓	0.68
*Osteichthyes*	0.71	↓	0.60	↓	0.50	↓	0.46	↓	0.57	↑	0.86	↗	0.72	↑	0.77	↓	0.05
*Mollusca*	0.76	=	0.76	↓	0.69	↓	0.33	↓	0.66	↗	0.78	↓	0.72	↗	0.78	↓	0.10
*Echinodermata*	0.79	↓	0.73	↓	0.69	↓	0.38	↓	0.74	↓	0.70	↗	0.77	=	0.79	↓	0.16
*Crustacea*	0.54	↓	0.49	↓	0.47	↓	0.21	↓	0.48	↗	0.57	↓	0.49	↗	0.55	↓	0.10
*Cnidaria*	0.79	↘	0.77	↓	0.73	↓	0.61	↓	0.71	↗	0.82	↓	0.75	↗	0.81	↓	0.37
*Bryozoa*	0.75	↘	0.74	↓	0.64	↓	0.54	↓	0.68	↓	0.67	↓	0.69	↓	0.71	↓	0.43
Arthropods	0.25	↓	0.17	↓	0.20	↓	0.11	↓	0.14	↑	0.29	↓	0.14	↓	0.21	↓	0.09
*Annelida*	0.57	=	0.57	↘	0.55	↓	0.36	↘	0.55	↗	0.59	↘	0.56	↗	0.59	↓	0.16
Avg	0.62	↘	0.59	↓	0.55	↓	0.41	↓	0.53	↗	0.63	↘	0.59	=	0.62	↓	0.24
Weighted Avg	0.77	=	0.77	↓	0.73	↓	0.59	↓	0.73	↗	0.78	↘	0.75	↗	0.78	↓	0.41

**Fig 7:**
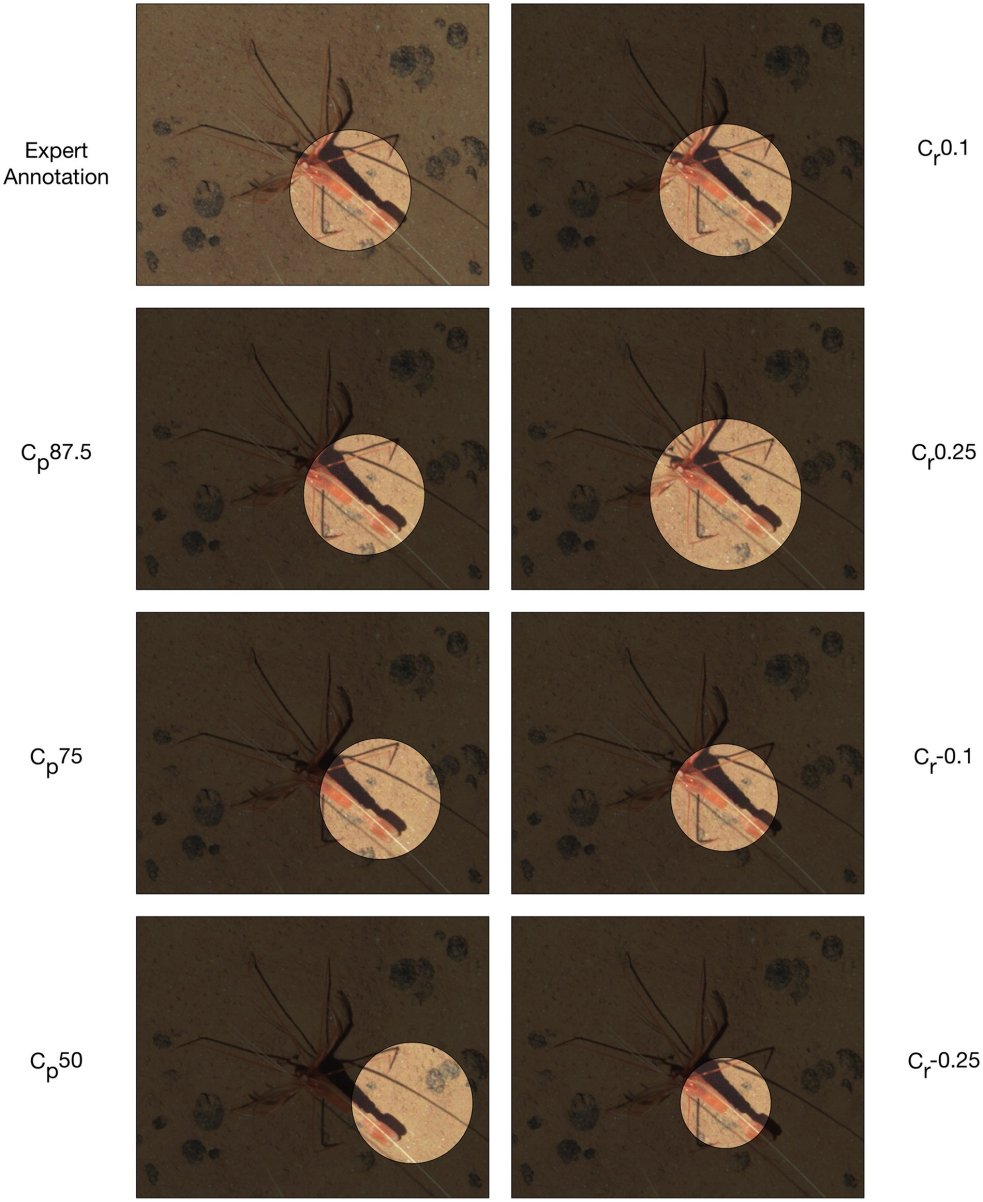
For illustration purposes we show the impact of different inaccuracies in position (IP) and radius (IR) on an annotation with the label *Crustacea*.

Our main interest lied on the question, to what extent the position and size of the CS annotation influences the classifier performance. Looking at the impact of the positioning inaccuracy (IP) on classification accuracy in Table 1 columns 2-4, we see a tendency that a decreasing minimum overlap yields a decreasing classifier accuracy. While *Protista* even gains 2 basis points when the minimum overlap changes to 87.5% due to IP, *Echinodermata* losses 6 basis points and *Osteichthyes* even losses 11 basis points. An explanation for this could be that *Protista* seem to have a unique texture everywhere, whereas *Osteichthyes* and *Echinodermata* have prototypical features at specific parts, e.g. the front and fins of *Osteichthyes* or the spines and the round shape of some *Echinodermata* (cf. Fig 2). When shifting the position from the center of the object to the periphery, this information can be partly lost or distorted. In Fig 7 (left column) this is illustrated for a *Crustacea*. When the minimum overlap decreases the classification results get worse. The classification results for *Annelida* and *Protista* are not affected as much as the other phyla (cf. Table 1 $C_{p}^{75}$).

Looking at the experiments where the radii of the annotation have been varied (cf inaccurate radius (IR), Table 1, columns 5-8) we observed an increase in classification accuracy for decreasing radii for most taxa. A larger radius seems to spoil the classifier performance. Although the overall accuracy decreases/increases, the per class accuracies behave quite differently, i.e. some classes seem to profit from the change while for others the accuracy drops. In contrast to the positioning inaccuracy (IP), the classifier appears to be more robust when the circle radius is changed (IR).

When looking at the classification results of $C^{CSP}$ (cf. Table 1, last column), we see a significant decrease in performance compared to the other experiments. However, this was somewhat expected because of the significant differences in the CS annotations compared to the expert annotations. Interestingly *Porifera* again only looses 14 basis points in classification accuracy in contrast to all other classes losing up to 66 basis points.

**Fig 8:**
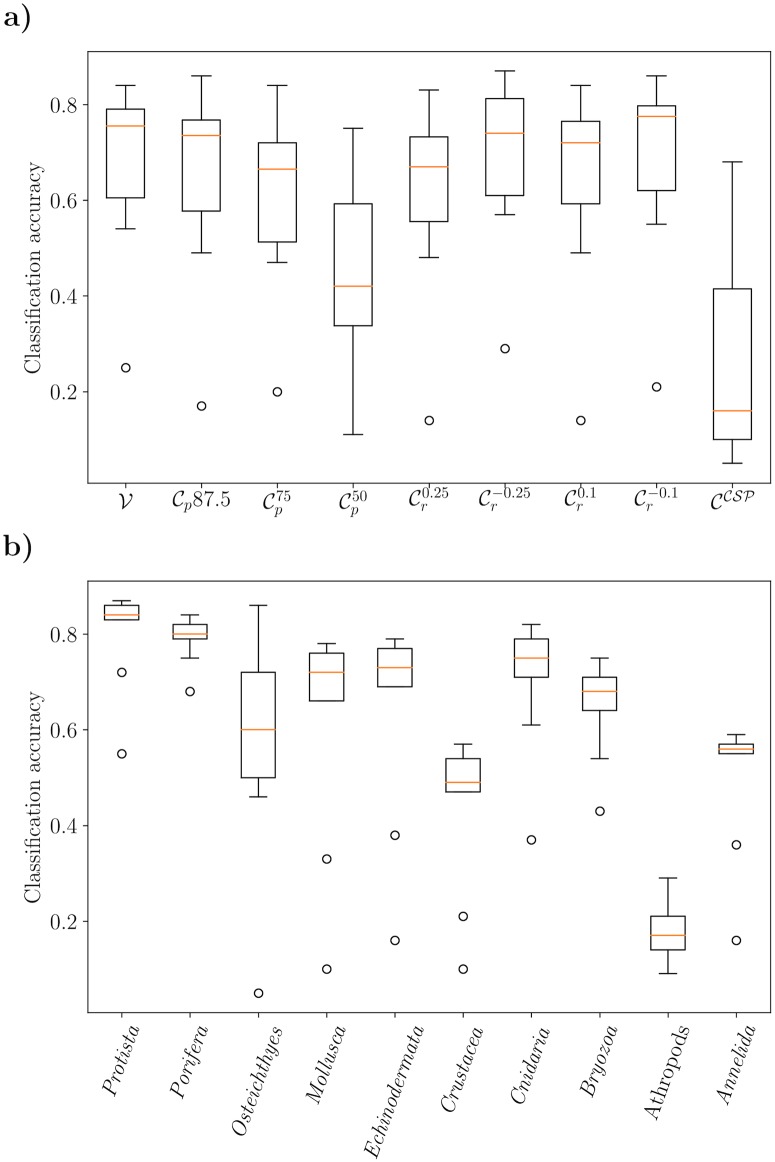
Classification accuracy for the expert, the simulated citizen scientist annotations, and the CS primer-experiment. a) Shows the accuracies aggregated by experiment and b) Each boxplot represents the aggregated results from each dataset ($V,C_{p}^{87.5},\ldots$) for each specific taxon.

The different taxa feature individual degrees of difficulty/robustness (see Fig 8) with Arthropods being the most complex classification task. We can also see that some taxa are more robust to changes than others in general. While *Protista*, *Porifera* and *Annelida* show only small changes, especially *Osteichthyes* show considerable variations in classification accuracy when changing the simulation parameters and thus the inaccuracies.

Although the experiments CSP and CSS show a quite high number of FP, these could be identified in the classification step and therefore compensated to some degree. The correct classification of FP as $f(C^{bg})=Background$ was performed with an accuracy of 96.78%.

## Discussion and conclusion

In our study, we have identified and analyzed the four common annotation parameters reflecting the differences between a CS derived annotation and an expert-derived one. Those are false positives (FP), false negatives (FN), inaccurate position (IP) and inaccurate radius/size (IR).

In the primer-experiment (eight citizen scientists detect objects in two images), we observed these problems and showed that large deviations, compared to expert annotations, can occur (IP and IR). As shown with the protocol-corrected expert annotations (cf. Fig 4c and 4d), these deviations mostly result from protocol differences, which could be minimized by providing the citizen scientists with more detailed instructions as shown in the CS study (CSS). In addition, the study showed that if a small crowd of untrained citizen scientists were assigned a rather complex task such as general object detection in marine images, a significant amount of data can be generated (CSP: 228 annotations CSS: 804 annotations), but not all data is meaningful, i.e. a lot of false positives (FP) are annotated. Furthermore, because of the complexity of the objects to be annotated, some objects are not found at all (FN).

We simulated datasets featuring different degrees of IP, IR and FP and used these as input to the deep learning classifier. We observed that incorrect radii (IR) are less severe than a position shift of the center with the correct radius (IP). Besides, some objects are more sensitive to deviations than others, most likely owing to their shape/structure. Objects incorrectly annotated as being interesting but belonging to a background class (FP) can be recognized with 96.78% accuracy by the classifier, which seems like a good enough value to compensate for this kind of error.

The above observations motivate the following recommendations for CS data collections in the context of machine learning applications:

1. CS should be provided with clear instructions and examples about the way the position and radius of the object should be marked.
2. The correct position seems more important than the correct radius.
3. Annotation radius should be selected not larger than necessary and should also be described by examples or explicitly defined for each taxon considered.
4. If CS annotations are used for classification, a smaller crop of the ROI can be tested for more accurate classifications.

The answer to the question of whether citizen scientists annotations are a feasible replacement for marine object detection in combination with deep learning classifiers has to be answered dichotomously. On the one hand, the results of the CS primer-experiment CSP suggest that if a significant divergence with expert annotations exists there is a considerable impact. This is underlined by the predictions of our CS simulations. On the other hand, if the deviations are not that huge like in the CSS, a minor impact in classification performance is noticeable.

Furthermore, measures to counter error-prone CS annotations using variations in the deep learning architecture or the training procedure, e.g. feeding simulated data to the classifier or increasing the size of the pooling layer might help in minimizing problems. For example, in this paper we showed that FP can be minimized by training the deep learning classifier to recognize background samples.

We conclude that if the CS study is well designed (see 1.-4. above, also cf. [7]), citizen scientist annotations are a valuable asset and errors can be within the limits of the other simulated data. Doing a joint annotation session or limiting the type of objects to be detected by a group of citizen scientists, thus splitting the task into object categories would also be useful to make the task more manageable for the citizen scientists and minimize errors. If this training is done thoroughly, it can be concluded that citizen scientist annotations are a compelling way to solve the marine object detection problem. Also, the same issues described here also arise when generating automatic detections and a similar conclusion can be drawn.
